# Exploring the Potential of a Behavior Theory–Informed Digital Intervention for Infant Fall Prevention: Mixed Methods Longitudinal Study

**DOI:** 10.2196/47361

**Published:** 2024-01-03

**Authors:** Nipuna Cooray, Catherine Ho, Amy Bestman, Susan Adams, Natasha Nassar, Lisa Keay, Julie Brown

**Affiliations:** 1 The George Institute for Global Health Faculty of Medicine and Health University of New South Wales Sydney Barangaroo Australia; 2 Department of Paediatric Surgery Sydney Children's Hospital Randwick Australia; 3 Children’s Hospital at Westmead Clinical School Faculty of Medicine and Health University of Sydney Sydney Australia; 4 School of Optometry and Vision Science Faculty of Medicine and Health University of New South Wales Sydney Sydney Australia

**Keywords:** child injury, digital behavior change interventions, user experience, falls, infant fall, injury, mobile app, digital intervention, users, mixed methods longitudinal study, behavior, development, fall risk, fall prevention, acceptability, app, children, internet, parents, maternal, paternal, accidents, infancy, infant, accidental fall, accidental falls, infant behavior, longitudinal design, mixed methods, parent, mobile phone

## Abstract

**Background:**

Falls are the most common hospitalized injury mechanism in children aged ≤1 years, and currently, there are no targeted prevention interventions. The prevention of falls in children of this age requires changes in the behavior of their caregivers, and theoretically informed digital behavior change interventions (DBCIs) may provide a unique mechanism for achieving effective intervention. However, user acceptance and the ability of DBCIs to effect the required changes in behavior are critical to their likelihood of success.

**Objective:**

This study aims to evaluate a behavior theory–informed digital intervention developed following a user-centered approach for user experience, the potential for this intervention to prevent infant falls, and its impact on behavioral drivers underpinning fall risk in young children.

**Methods:**

Parents of infants aged <1 year were recruited and asked to use the intervention for 3 months. A pre-post longitudinal design was used to examine the change in the potential to reduce the risk of falls after a 3-month exposure to the intervention. Postintervention data on behavioral drivers for fall prevention, user acceptability, and engagement with the app were also collected. Interviews were conducted to explore user experiences and identify areas for further improvement of the intervention.

**Results:**

A total of 62 parents participated in the study. A statistically significant effect on the potential to reduce falls was observed after the intervention. This effect was higher for new parents. Parents agreed that the intervention targeted most of the target behavior drivers. The impact of behavior drivers and intervention on the potential for fall prevention had a positive correlation. The intervention demonstrated good levels of acceptability. Feedback from participants was mostly positive, and the primary area identified for further improvement was widening the scope of the intervention.

**Conclusions:**

This study demonstrated the promise of a newly developed digital intervention to reduce the risk of infant falls, particularly among new parents. It also showed a positive influence of the DBCI on the drivers of parental behaviors that are important for fall reduction among infants. The acceptability of the app was high, and important insights were gained from users about how to further improve the app.

## Introduction

Children aged ≤1 year, that is, infants, have the highest rate of death owing to fall-related injury, and falls are the most common injury mechanism resulting in emergency department visits and hospitalizations during infancy. The head is the most commonly injured body part owing to infant falls [1-5], and in severe cases, these result in skull fractures, traumatic brain injuries, and long bone fractures [6].

Most of these fall events can be prevented by age-appropriate safe parenting practices and making changes in the child’s environment [7-9], but currently, there are no targeted, proven interventions specifically for infant fall prevention [10,11], and there is also evidence that fall injuries have increased in recent years [12].

To fill this gap, the research team created a behavior theory–based digital intervention for infant fall prevention following an iterative user-centered process [13]. As detailed in the first paper, the Behavior Change Wheel (BCW) [14] combined with the person-based approach [15] was used to theoretically inform and develop a user-centered digital intervention. The resulting intervention included 4 modules targeting common fall mechanisms and events occurring within the first year of an infant’s life.

The four modules consisted of (1) a safe feeding module targeting the prevention of falls related to feeding, (2) a safe furniture use module targeting infant falls related to furniture, (3) a safe use of baby products module targeting infant falls related to baby products, and (4) a safer environment module targeting stairs-related infant falls. The main features of the app include written articles (13 short articles, reading time per article approximately 3-5 min), trackable “tasks” encouraged by the articles where users can check off tasks as they complete them, a dashboard allowing users to check adherence with suggested tasks, and push notifications to remind users to engage with the app. (see the app screenshots in Multimedia Appendix 1).

The user-centered approach taken to develop this intervention inherently focused on ensuring that the target population comprehended the material provided and that the method of delivery was acceptable to users. However, as detailed in the first paper [13], this development process was undertaken iteratively with each individual module, and there is a need to ensure the acceptability of the overall app that integrates the 4 modules. Although the use of the BCW in designing the app was intended to increase the likelihood that engagement with the app would lead to the adoption of behaviors required to reduce the risk of falls among infants, this is not guaranteed, and there is also a need to evaluate whether the app is likely to have the desired impact and if this impact is consistent across all users. Finally, the app can only realize its desired effect if there is appropriate engagement by users with the app, and there remains the need to assess likely parental engagement and the scope for further improving engagement. This paper presents a 3-month longitudinal study to address these questions. The specific aims of this study were as follows:

1. To determine the overall impact of exposure to the intervention on parents’ potential to reduce the risk of infant falls and determine if this is consistent across all users.
2. To examine the behavioral drivers for falls prevention (capability, opportunity, and motivation) among parents after exposure to the intervention and examine the relationship between these factors and the impact of the intervention.
3. To determine acceptability of the app as a whole and engagement with the app.
4. To explore user experience to identify factors driving user acceptability and engagement and scope for further improving the intervention.

## Methods

### Study Design

This study used a pre-post longitudinal design to examine the change in the potential of parents to reduce the risk of falls after a 3-month exposure to the intervention (part 1). The postexposure survey delivered at 3 months also collected data on behavioral drivers for falls prevention (part 2), user acceptability, and engagement with the app (part 3). User experience was further studied through in-depth interviews with a subset of participants to provide insight into factors driving user acceptability and engagement and to identify the scope for further improvement of the intervention (part 4). Parts 1 to 3 were quantitative and part 4 was qualitative. A mixed methods analysis approach was then used to triangulate the user experience findings from parts 3 and 4.

### Ethics Approval

Ethics approval for the study was obtained from the University of New South Wales Human Research Ethics Executive Committee (HC210494).

### Study Setting and Participants

Inclusion criteria for participants taking part in this study were as follows: must be aged ≥18 years, a parent of a child aged 0 to 6 months or an expectant parent within 2 months of the due date (mother or father), living in Australia, able to speak and understand English, and have access to a smartphone (iOS or Android). The study duration was 3 months. Participants needed to be in Australia with access to an iOS or Android smartphone because of the availability of the app in relevant app stores. The study duration was selected to cover the relevance of the information within the intervention.

### Recruitment

Participants were recruited from an Australian market research company’s existing consumer panel of parents between November and December 2021. A screening survey with questions to assess the inclusion criteria was emailed to the members of the consumer panel. Eligible participants who registered their interest in participating were electronically sent the main consent form to read and consent.

Within the main consent form, participants were invited to opt-in for the in-depth interviews, but a decision not to opt-in to this component did not preclude involvement in the main study. The first 10 consenting participants who opted in and provided separate consent for the in-depth interviews were selected.

The participants were given a gift voucher for Aus $100 (US $65) for completing both baseline and poststudy surveys. In addition, participants who took part in the poststudy in-depth interviews were given an Aus $40 (US $26) gift voucher.

### Access to the App

Participants were provided with a link to download the app from the Google Play Store or Apple App Store depending on the smartphone they own.

### Data Collection

Baseline and poststudy quantitative data were collected via a survey hosted on REDCap (Research Electronic Database Capture; Vanderbilt University) and distributed to participants electronically (Table 1). The baseline survey collected data on participant demographics (such as education level, income level, number of children, and marital status; Table 2) and questions designed to measure participants’ potential to reduce the risk of infant falls. The latter consisted of the following four questions:

1. I know how to prevent falls among young children
2. Falls in children aged ≤1 year can be prevented
3. I am confident I can take actions to reduce the risk of my child falling
4. I have taken specific actions to reduce the risk of my child falling

The same 4 questions were also included in the poststudy survey. This set of questions was designed to demonstrate whether exposure to the app had the overall desired impact and was measured using a 5-point Likert scale (strongly disagree to strongly agree). The poststudy survey also included open-ended questions designed to (1) collect data from participants on the behavioral drivers for potential to reduce the risk of infant falls (Table 3) based on the Capability, Opportunity, Motivation–Behavior (COM-B) self-evaluation questionnaire [16] and (2) collect information on user experience. The latter user experience questions were framed in terms of user acceptance and engagement with the app.

User acceptance was measured by asking participants how much they liked the app and to rate the level of agreement (Likert scale: strongly disagree to strongly agree) with the following six statements: (1) I found the app easy to use; (2) I found the information useful; (3) the advice provided was easy to follow; (4) I could act on the advice provided; (5) I like the features of the app; (6) I found the reminders or notifications helpful.

For engagement, participants were asked about their use of the app and its features and to respond to the statements “I used the app” (not at all; once; more than once but not often; often—more than once a month; frequently—more than 4 times a month); “I read all the articles” (Likert scale: strongly disagree to strongly agree); and “I used the task list feature” (Likert scale: strongly disagree to strongly agree).

Poststudy qualitative interviews were conducted with a subgroup of 10 participants to understand parents’ user experience with the app and to understand further opportunities to improve the app. In-depth interviews were conducted face-to-face using videoconferencing (Microsoft Teams). A discussion guide was used to structure interviews with each participant. The discussion guide was developed to ensure that participants understood the context of the discussion and to collect more in-depth details about the factors driving their acceptability and engagement with the app than could be collected through a quantitative survey. Similarly, it was also designed to collect more detailed insight into how the material provided in the app influenced the behaviors required to reduce the risk of falls in infants. Before conducting the interviews, the discussion guide was refined through peer-to-peer testing to optimize discussion flow and clarity. The final discussion guide used to frame the 10 in-depth interviews is provided in Multimedia Appendix 2. All the interviews were recorded and transcribed verbatim.

**Table 1:** Summary table of research variables.

Aim, variable, and source			Data type	Analysis conducted
**Aim 1**				
	**Factors assumed related to fall prevention (secondary outcome)**			
		Response to survey questions: I know how to prevent falls among young children (a)	5-point Likert scale	Difference between before and after using a paired Wilcoxon signed rank test
		Falls in children under 1 can be prevented (b)	5-point Likert scale	Difference between before and after using a paired Wilcoxon signed rank test
		I am confident I can take actions to reduce the risk of my child falling (c)	5-point Likert scale	Difference between before and after using a paired Wilcoxon signed rank test
		I have taken specific actions to reduce the risk of my child falling (d)	5-point Likert scale	Difference between before and after using a paired Wilcoxon signed rank test
	**Potential to reduce falls (primary outcome)**			
		Sum of a to d	Continuous variable	Difference in means before and after using a 1-tailed paired *t* test
	**Intervention impact (“total change”—primary outcome)**			
		Calculated by postintervention potential to reduce falls minus preintervention potential to reduce falls	Continuous variable	Difference in means between different demographic groups using a 1-tailed paired *t* test
	**Demographics (independent variable)**			
		Various levels (Table 2) for relationship to child, age, experience, country of birth, household income, marital status, and education level	Categorical variable	Difference in means between different demographic groups using a 1-tailed paired *t* test
**Aim 2**				
	**Intervention impact (“total change”—primary outcome)**			
		Calculated by postintervention potential to reduce falls minus preintervention potential to reduce falls	Continuous variable	Linear regression used to examine influence of behavior scores on intervention impact while controlling for parental experience
	**Capability score (independent variable)**			
		Response to survey questions (Table 3) by Likert scales summed	Continuous variable	Linear regression used to examine influence of behavior scores on intervention impact while controlling for parental experience
	**Opportunity score (independent variable)**			
		Response to survey questions (Table 3) by Likert scales summed	Continuous variable	Linear regression used to examine influence of behavior scores on intervention impact while controlling for parental experience
	**Motivation score (independent variable)**			
		Response to survey questions (Table 3) by Likert scales summed	Continuous variable	Linear regression used to examine influence of behavior scores on intervention impact while controlling for parental experience
	**Overall behavior score (independent variable)**			
		Calculated by the aggregate of capability, opportunity, and motivation scores	Continuous variable	Linear regression used to examine influence of overall behavior score on intervention impact while controlling for parental experience
	**Experienced parent (confounder)**			
		Yes=2 or more children; no=1 child	Categorical variable	Linear regression used to examine influence of overall behavior score on intervention impact while controlling for parental experience
**Aim 3**				
	**Engagement (outcome)**			
		Response to survey questions	5-point Likert scale	Descriptive statistics
	**Likeability (outcome)**			
		Response to survey questions	5-point Likert scale	Descriptive statistics
**Aim 4**				
	**Barriers and enablers of the intervention**			
		Poststudy interview	Qualitative data	Qualitative descriptive method

**Table 2:** Participant demographics (N=60).

		Participants, n (%)	Total change, mean (SD)	*P* value	
**Relationship to child**					.48
	Mother	54 (90)	2.35 (2.17)		
	Father	6 (10)	1.67 (2.80)		
**Age (y)**					.41
	26-35	40 (67)	2.15 (2.34)		
	36-45	19 (32)	2.68 (1.97)		
	46-55	1 (2)	N/A^a^		
**Number of children (dichotomized to a new parent and experienced parent)**					.03
	0 (new parent)	20 (33)	3.15 (2.30)		
	1 (experienced parent)	40 (67)	1.85 (2.08)		
**Parent born in Australia**					.57
	Yes	49 (82)	2.20 (2.26)		
	No	11 (18)	2.64 (2.11)		
**Household income (Aus $)**					.48
	<Aus $ 100,000 (<US $65,000)	15 (25)	1.81 (1.87)		
	Aus $ 100,000-Aus $ 150,000 (US $65,000-97,500)	20 (33)	2.40 (2.50)		
	≥Aus $ 150,000 (≥US $97,500)	21 (35)	2.29 (2.33)		
	Decline to answer	3 (5)	4.00 (0)		
**Marital status**					.99
	Married	43 (72)	2.28 (2.26)		
	Single parent	3 (5)	2.33 (2.52)		
	De facto (common law marriage)	14 (23)	2.29 (2.23)		
**Education level**					.66
	Primary school, secondary school, and some university or TAFE^b^ diploma	20 (33)	2.20 (1.88)		
	University or TAFE graduate	25 (42)	2.08 (2.38)		
	Postgraduate degree	15 (25)	2.73 (2.46)		

^a^N/A: not applicable.

^b^TAFE: technical and further education.

**Table 3:** The mean level of agreement with intervention impact on behavioral drivers.

		Values, mean (SD)
**The app has improved my knowledge on (capability)**		
	The importance of getting rest	3.93 (0.94)
	How to reduce fall risk while feeding my baby	4.15 (0.84)
	How to reduce fall risk while my baby sleeps	4.12 (0.90)
	How to reduce fall risk while changing my baby	4.40 (0.79)
	How to reduce fall risk when using baby products like chairs and prams	4.17 (0.85)
	How to reduce fall risk on stairs	4.30 (0.79)
	Overall	4.18 (0.86)
**After using the app, I feel (opportunity)**		
	I have the support I need to get enough rest	3.50 (0.98)
	I have everything I need to reduce fall risk while I feed my baby	4.35 (0.60)
	I have everything I need to reduce fall risk while my baby sleeps	4.38 (0.56)
	I have a safe place to change my baby	4.52 (0.68)
	I am able to correctly use safety straps when using baby products like chairs and prams	4.58 (0.53)
	I have everything I need to reduce fall risk on stairs	4.20 (0.73)
	Overall	4.26 (0.78)
**After using the app (motivation)**		
	Remember to ask for help when feeling tired and feeding my baby	3.90 (1.00)
	Have established a routine to reduce fall risk while feeding my baby	4.08 (0.81)
	Intend to ensure my baby always sleeps in a cot	3.95 (1.17)
	Believe changing my baby on the floor is the best option if I do not have access to a safe change table	4.53 (0.77)
	Have established the habit of correctly using safety straps when using baby products like chairs and prams	4.42 (0.74)
	Believe stairgates are important in areas accessed by my child	4.68 (0.50)
	Overall	4.26 (0.90)

### Analysis

The R programming language (R Foundation for Statistical Computing) was used for statistical analysis. In-depth interview data were transcribed and analyzed using NVivo software (Lumivero). Sample characteristics for the 60 participants in the longitudinal study were examined using descriptive statistics. The analytical approaches varied for each part (parts 1-4) of the study. The following section describes the approach adopted for each part.

### Part 1: Determining the Overall Impact of Exposure to Intervention to Reduce the Risk of Falls

The primary outcomes studied in part 1 were the change in responses to the 4 questions included in both the pre- and poststudy surveys (ie, I know how to prevent falls among young children; falls in children under 1 can be prevented; I am confident I can take actions to reduce the risk of my child falling; and I have taken specific actions to reduce the risk of my child falling) and change in overall participants “potential to reduce the risk of infant falls.” The latter was calculated from both pre- and poststudy responses by summing the Likert values for each of the 4 questions. An “intervention impact” score was then calculated by subtracting the total pre score from the total post score.

The pre-post difference in responses to the 4 questions was examined using paired Wilcoxon signed rank tests. The pre-post difference in the overall “potential to reduce the risk of infant falls” was examined using a 1-tailed paired *t* test.

The influence of exposure to the app on potential to reduce the risk of infant falls for different types of participants (as described by demographic variables: relationship to child, age of parent, 1 or more children, country of birth, household income, marital status, and education level) was examined by testing the difference in mean “intervention impact” between the different demographic groups. For dichotomous variables, independent 1-tailed *t* tests were used, and for variables with ≥2 category levels, ANOVA was used.

### Part 2: Behavior Drivers for Fall Prevention After Exposure to the Intervention and the Relationship Between Behavior Drivers and the Impact of the Intervention

To examine the behavioral drivers for falls prevention (capability, opportunity, and motivation) among parents after exposure to the intervention, the mean level of agreement with each of the capability, opportunity, and motivation statements (Table 3) was calculated across the whole sample, together with the overall mean for each group of statements across the whole sample, that is, a mean overall capability, opportunity, and motivation score.

To examine the relationship between the behavioral drivers as self-evaluated by participants and the impact of the intervention, a capability, opportunity, and motivation score was calculated for each participant by summing the level of response provided for each question in each group (Table 3), and an overall “behavioral driver” score for each participant was calculated by summing the level of agreement with each statement listed in Table 3. The association between the behavioral component scores (ie, capability, opportunity, and motivation scores for each participant) and the impact of the intervention was examined using multivariable linear regression. A second linear regression analysis was then conducted to examine the relationship between the overall behavioral driver scores and the impact of the intervention. In both regression models, demographic variables found to be significantly associated with the impact of the intervention were also controlled for parent’s experience.

### Part 3: Determining Acceptability of the App as a Whole and Engagement With the App

Engagement with the app, as measured using responses to the question “I used the app,” and the number of tracked tasks per participant were examined using descriptive statistics.

The acceptability of the app as a whole was determined by calculating the mean levels of agreement for each “app-like” statement across the sample, together with the mean overall level of agreement for this group of “app-like” statements across the sample.

### Part 4: Explore User Experience to Identify Factors Driving or Hindering User Acceptability and Engagement and Scope for Further Improvement

The in-depth interview data were analyzed using a qualitative descriptive method to identify barriers and enablers of the intervention in terms of user experience [17].

### Sample Size

A sample size of 62 was estimated to be sufficient for the quantitative components of the study based on a power calculation to see a significant change in parents’ potential to reduce the risk of falls with an effect size of 0.4 and 80% power at the 5% level, allowing for up to 20% loss to follow-up and rounding up to the next full number.

For the qualitative poststudy in-depth interview, a sample size of 10 was chosen using a rule of thumb that this sample size should be sufficient to reach saturation and is double the minimum sample size recommended for digital intervention usability studies with a sample of 5 [18].

## Results

### Sample Characteristics

A total of 62 participants were recruited, downloaded the app, and completed the baseline survey, with 2 (3%) lost to follow-up. Therefore, 60 participants completed the poststudy survey. Table 2 presents the sample characteristics. In summary, 54 (90%) were mothers, 40 (67%) were aged 26 to 35 years, 20 (33%) were new parents, and 49 (82%) were born in Australia; 43 (72%) patients were married.

### Part 1: Determining the Overall Impact of Exposure to the Intervention to Reduce the Risk of Falls

**Figure 1:**
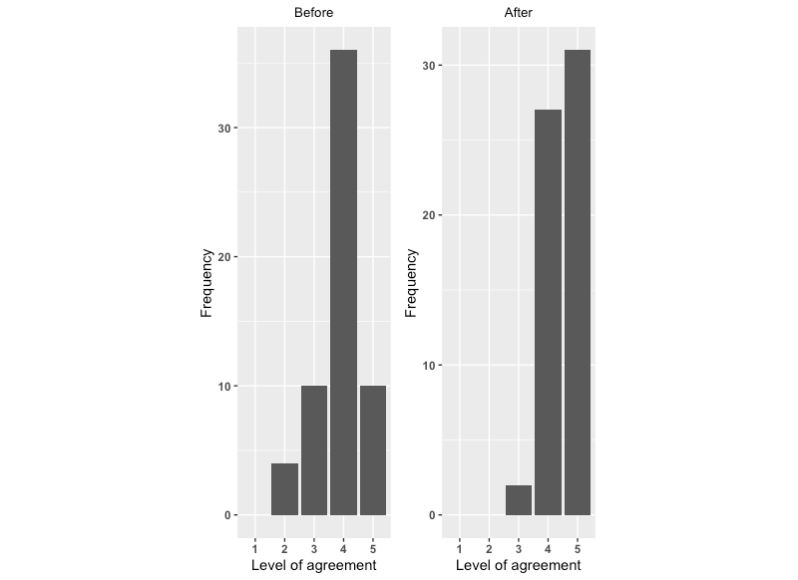
Level of agreement with “I know how to prevent falls among young children”: before versus after.

**Figure 2:**
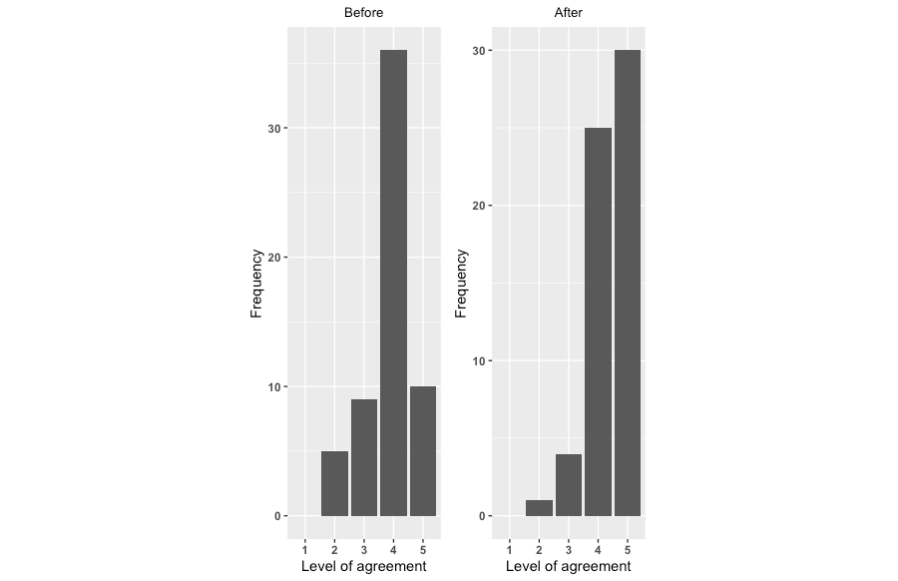
Level of agreement with “Falls in children under 1 can be prevented”: before versus after.

**Figure 3:**
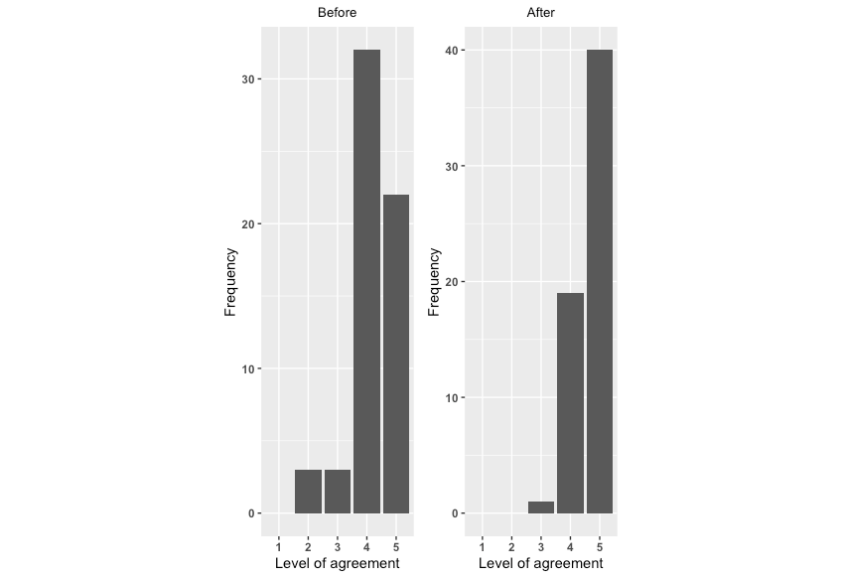
Level of agreement with “I am confident I can take actions to reduce the risk of my child falling”: before versus after.

**Figure 4:**
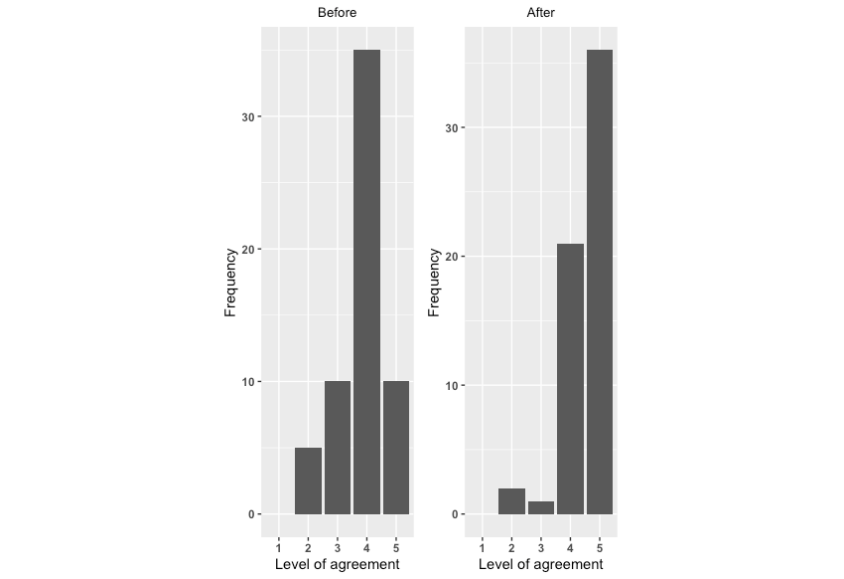
Level of agreement with “I have taken specific actions to reduce the risk of my child falling”: before versus after.

There was a significant improvement in each measure of the potential to reduce the risk of infant falls from after exposure to the intervention compared with before exposure. For each question, there was a significant increase in the level of agreement with the statements (Figures 1-4; *P*<.001).

There was also a significant improvement in the overall potential of parents to reduce the risk of falls after using the intervention. The mean overall score among participants before exposure was 15.77 (SD 2.24; range 10-20) and 18.05 (SD 1.86; range 14-20) after a 3-month exposure to the app (*P*<.001). Across the entire sample, the mean “total change” in potential to reduce the risk of falls was 2.28 (SD 2.23; range −2 to 8).

Table 2 presents the “total change” according to the different participant demographics. The only significant difference by demographics was a significantly greater “total change” in the potential to reduce the risk of infant falls among participants with only 1 child. Parents with ≥2 children had a mean “total change” of 1.85 (SD 2.08; range –2 to 5) whereas the less-experienced parents with only 1 child had a mean “total change” of 3.15 (SD 2.30; range –1 to 8; *P*=.03).

### Part 2: Behavior Drivers for Fall Prevention After Exposure to the Intervention and the Relationship Between Behavior Drivers and the Impact of the Intervention

The mean level of agreement with each statement across the sample is shown in Table 3. As shown in Table 3, there was strong agreement with overall capability, motivation, and opportunity as self-evaluated by participants after using the app. The only aspect where there was inconsistent strong agreement was in questions related to getting enough rest where capability, opportunity, and motivation means across the sample remained <4.

Tables 4 and 5 present the results of the linear regression analyses. In the univariate analysis, opportunity and motivation scores were significantly associated with the impact of the interventions, with increasing behavioral component scores associated with increasing impact scores. However, in the multivariable analysis when controlling for parent experience (which was found to be significantly associated with the impact of intervention in part 1), none of the individual behavior components were significantly associated with impact (Table 4).

As shown in Table 5, the overall behavior score was significantly associated with increasing impact scores, even when controlling for parents’ experience.

**Table 4:** Regression analysis modeling the relationship between behavior drivers and intervention impact.

	Univariate			Multivariable	
	Estimate	*P* value	Estimate		*P* value
Capability score	0.127	.07	−0.017		.86
Opportunity score	0.257	.006^a^	0.191		.12
Motivation score	0.207	.01^a^	0.926		.49
Experienced parent (yes or no)	−1.3	.03^a^	−1.196		.04^a^

^a^*P*<.05.

**Table 5:** Regression analysis modeling the relationship between overall behavior score and intervention impact.

	Univariate		Multivariable	
	Estimate	*P* value	Estimate	*P* value
BD^a^ score	0.079	.009^b^	0.075	.01^b^
Experienced parent (yes or no)	−1.3	.03^b^	−1.118	.04^b^

^a^BD: Behavior Drivers Score.

^b^*P*<.05.

### Part 3: Determining Acceptability of the App as a Whole and Engagement With the App

Table 6 presents the mean level of agreement of app use statements across the sample of participants, including their app use, whether they read the articles, and used the task tracking feature. The mean number of completed tasks per participant was 24 (SD 24.2052).

Table 7 presents the mean levels of agreement across the whole sample for each of the “app-like” statements and the mean overall, indicating generally strong acceptability (agreement levels over 4) of the app as a whole. The lowest levels of agreement were in the response to the “I like the features of the app” and “I found the reminders/notifications helpful.”

**Table 6:** Participant agreement with the use of the intervention.

App use	Scores, mean (SD)
I used the app	3.53 (0.68)
I read the articles	4.17 (0.98)
I used the task tracking feature	3.75 (1.31)

**Table 7:** Participant agreement with the acceptability of the intervention.

App-like	Scores, mean (SD)
I found the app easy to use	4.20 (0.71)
I found the information useful	4.05 (0.79)
The advice provided was easy to follow	4.52 (0.60)
I could act on the advice provided	4.22 (0.94)
I like the features of the app	3.67 (0.93)
I found the reminders or notifications helpful	3.68 (0.89)
Overall score	4.05 (0.87)

### Part 4: Exploring User Experience to Identify Factors Driving or Hindering User Acceptability and Engagement and Scope for Further Improvement (Qualitative Analysis)

#### General Understanding of Child Injury and Feedback on the Intervention

Parents expressed that the intervention provided them with important information, which was new. The intervention seemed to be valued more by new parents. It was evident that child injury was not an area that parents gave much attention to (“Injury is not something I had put too much thought into, I guess I just considered a baby didn’t move”). Some parents had a general understanding and personal fear about falls due to the experiences of other parents they knew. In addition, safe sleeping practices and safe change table practices were commonly identified as information parents received from antenatal classes (“one of the main ones that I sort of looked at when my first son was born was more pages like SIDS”). However, the consensus was that they had little knowledge of specific preventive actions for most of the common fall events:

I learned so much...it brought to light a lot of things that you wouldn’t think of...first I was like, you know, but then every time I would read something, I’d learn something.

Experienced parents (who had more than 1 child) also identified the importance of the intervention. Even in cases where they knew some preventive actions beforehand due to lived experiences (“it was really just some anecdotal stories of my friends babies falling off things and hurting themselves...”), they identified that the intervention was a good reminder to adhere to safe behaviors:

I can’t remember how I learned the information with my first child, but it was definitely a great reminder and easy access to find the information all in one place.

Parents liked the style of the articles, which they reported they found engaging and informative and liked that the length was not too long. They also identified that if articles were too long, parents may not bother reading them, particularly as during the first year of a child’s life a lot of information is “thrown” at parents:

I thought it was really well written. I liked the style of the way that it was written. I thought it was. It was very engaging and informative and I liked the length of it as well. But it wasn’t too long. It just made it easy to when you’re busy as a mum, sort of dip in and out of just having a bit of a look and yeah, and like getting some information quite quickly rather than reading pages and pages out. It was also quite easy to access different topics with them.

Parents also identified the importance of the tone of the intervention and appreciated the practical nature of the advice provided:

I liked it because it was really straightforward. It’s not in any way condescending. I don’t think it’s like I think sometimes you read resources and they can be like talking down to you. But I found that it was like simple language, but not in a condescending way.

Credible profiles were valued by parents. They requested more ways to show the credibility of the information such as embedded links below the articles from reputable organizations:

And I like it. It’s got the...like professors on there.... So it’s telling you that there’s experts on this.

The task-tracking and adherence dashboards were features liked by most participants. “Checking things off a list” was well liked. The adherence dashboard was found to be an incentive “to get it all green.” Parents understood the rationale of task tracking and expressed its importance in encouraging them to adhere to what was conveyed in the articles. However, there were some issues with the user experience of this feature (“I’m not finding easy to find what the ongoing ones are”). One parent requested access to the task list directly, without going through any related article. Another expressed that they could not get “100%” due to some tasks being not relevant for them. The “if-then” tasks were somewhat disliked:

I think that I think that idea of it is good, like having tasks that you can go through and say, yes, we’ve done this, but just the way it was delivered.

I quite like checking things off. At least you know this is quite satisfying to kind of get to the ending. Right. OK, well, you know, I have half a clue about what I’m doing in this area.

The notifications were found to be helpful. Parents expressed that the notifications made them come back to the app, made them read the articles, and helped them adhere to suggested practices. However, 2 participants mentioned that they did not receive notifications:

I liked the little reminders and I do. I do like that ‘cause. I think you get busy and then you don’t think to use it. So I did like the notifications as a way from reminding to dip back in and have information.

App esthetics was also positively received. Parents liked that the app used real photos rather than drawings. Several “typos” within the content were noticed by the parents and were negatively received (“I’m a bit of a stickler for, you know, the text, I guess so, like typos and things, you know”).

#### Feedback on 4 Modules

Information provided in the safe feeding module was not practical for some parents. This was because it was dependent on the amount of social support available for mothers.

In addition, some mothers identified the possibility of cosleeping occurring, although this was not suggested by the module. One mother indicated that her initial evaluation of the intervention was not positive because of this inapplicability to her situation:

Uhm, I think probably this one was the least effective for me. I think like I like I mean it. I thought it was very helpful for reminding, reminding you of the importance of rest in that, that there it is risky to feed while you are tired. I think like in terms of actually making changes, it was I think the app was less helpful in doing that then the other modules because it was really around like getting support and that sort of thing, which is something that the app can’t necessarily help you with because either you have social support and you have people that can help you or you don’t work down the other modules provided more practical advice which weren’t sort of dependent on social support.

Get enough sleep, you know, have your partner or someone to support you to come and do that? Well,... I actually left my job after my first one, and my husband still works and he gets up at 4:00 AM. It’s unrealistic for me to expect that. But for me, the one who doesn’t work to say, Oh no, you need to get up. Or every second night you’re on duty to take over. It’s just not possible.

Some advice was not practical for parents with more than one child, such as “sleeping while the baby sleeps,” where they have to take care of the other child at the same time (“to sleep when the baby sleeps, I thought, what about my toddler, he doesn’t nap”).

Despite these limitations, parents understood the reasons for the suggested practices. They also expressed that the module made them conscious when they were tired during night feeding and encouraged them to take action to reduce the chance of falling asleep while holding the baby. Some expressed the importance of acknowledging differences in individual experiences within the module to mitigate any negative feelings induced (“it’s one of those things where it’s like ideal situation, right. But the reality is just sometimes so different”).

The parents liked the safe furniture use module. The message “one day the baby is still, and next day they are rolling,” resonated well with the parents. Commonly, parents expressed that they practiced keeping the baby in the cot if they had to move away or “on the floor,” where they could not fall, but some parents still left their baby on a bed, when they are in the prerolling age, in the “middle of the bed”:

I really liked this one. It made me yeah, it really made me reconsider that. Uhm, you know, even if you think either in the middle of the bed they are fine, that actually like it, they made that maybe when they roll and so it may be this. So this was helpful for me in thinking actually, although you’re tempted to put them on the bed or put them on the change table, they’re actually safer on the floor like putting them on the carpet actually safer than putting them on the bed. So this one actually really did stick with me. And that’s something that I thought about continually is actually just put him on the floor and he’s safer ’n the floor 'cause he can’t. fall anywhere.

Parents found safe nappy change practices to be acceptable when using a change table. For some, this was aligned with the information received previously in the antenatal classes, but most mentioned that there are no safety straps in their own change tables, so keeping a hand on the baby was the applicable advice (“I don’t have a change table with straps and I do. I’m not sure that that’s a standard thing”). Changing nappies on the floor “where they can’t fall” was also liked by some, but some expressed this may be not practical in instances where the mother had a cesarean birth (“but as a mom who had two caesareans, I’m not gonna be getting down on the floor with a newborn baby”).

The module for the safe use of baby products was well received. Parents reported that the module made a difference on how they used safety straps with products. Some parents previously did not think of using straps when the baby was “very small” but reported that the module had an influence on changing the practice of using safety straps. One parent found that the information also influenced how they picked secondhand baby products, which seemed to be a common practice:

I think it might has made me so that you always say the straps here, but I think it’s just made it. It really reinforced to me how important it is to always do it up and if they look, you know that you think are there sitting there. They look secure, already without the straps on just to make it a habit of doing them up. So that was really good reminder for me that or you always need to just do it up just to just for that safety ‘cause you never know when they’re gonna try and reach for something or rollout or slide out. I think it also made me more aware of When I was buying secondhand baby items, they saw that all of the clips and everything were usable and that and present.

The use of a wheeled baby walker was well accepted. Parents had commonly received some information on the negatives of baby walkers before the intervention. They understood that wheeled walkers are a fall risk and might also affect a baby’s natural ability to stand. One parent mentioned that they still used one, but the duration of use was reduced after the intervention:

No, I never did. Somewhere I read early on that they aren’t safe ‘cause they can get to places they shouldn’t be able to get to. Uh,.... So I’ve never had one. I had a bouncer that didn’t move. That’s probably not great for their development, but in put them in it too much. But it wasn’t their safety thing in terms of, yeah, getting places, they shouldn’t get to.

Parents who had stairs in their homes identified the importance of the information included in the module for creating a safe home. Parents knew about using safe gates but identified the importance of other stair safety practices, but commonly, parents who did not have stairs found this module not relevant to them. They requested relevant information, such as babyproofing the environments:

We already knew that we needed to get safety gates. I’ve got friends with children. So you’re already aware of the gates. But I do find the app. Yeah. Helpful from just from the tips around stairs. So, like, sort of saying don’t step over the gate. And that’s really stuck out to me. Was like, hold onto the rails and like and making sure that you have a free hand because it’s so easy with stairs to hold baby in one hand and then be carrying a cup of tea or something else with the other hand. That is something that has changed my behavior, like making sure that I don’t have my hands full and nothing to hold onto.

#### General Feedback

Most of the interviewed parents expected more from the intervention. Even those who really liked the intervention expected more. Some thought the scope was too narrow, focusing only on falls, compared with multiple injury mechanisms, and mentioned that they may not have used it if they came across it outside the study. In addition, they felt that the intervention should have more engaging features (“It need something to keep you coming back to”). Parents had several suggestions to improve the scope of the intervention, such as providing more information relevant to older children, information on other child injury types, and prevention (babyproofing the house), including first-aid information and tapping into other relevant early childhood information:

...And I know this is sort of more a pilot, but I just wanted to see more, but I think that’s where you’re going with it. I’m, I’ve got through the modules quite quickly and I thought there’s no more. I finished it now.

...but I would really love like a checklist of this is everything that you need to do, you know, to baby proof your house so, you know, draw locks, baby gates like a, you know, like a a nice little comprehensive list for you to sort of do a scan of your house and then, everything you can do to make it injury safe, your baby.

Parents expressed the importance of receiving the app from reputable agents to find it valuable and for them to use it (eg, via an antenatal class):

I think if I’d been aware of it, yeah, I definitely think so. So yeah, if, yeah, if at the hospital or the midwife or if it had been in, you know, the baby bundle that you get if there’d been a little flyer. Yeah, it would be something I’d look at. And definitely if I’d known it.

Parents reported that they liked mobile apps rather than scanning through websites to obtain relevant information. Similarly, it was evident that although they tended to use social media groups (Facebook groups) to seek childcare information, they preferred reputable sources and sources where they can find professionally backed reputable information:

And sometime like when you’re looking at websites and stuff, it can get so confusing, whereas like having an app or just one place to look just makes things so much more straightforward.

I’ve recently got rid of Facebook because they’re or maybe within those groups, there tends to be lots of negativity and scaring and I would be, I think it’s taken me three kids and this long to realize that it’s probably not a space I really want to be in and without an expert moderator, I don’t wanna be there ‘cause you can get too much information.

Parents felt that there is a place for digital interventions in the space of early childhood interventions. Several parents shared the opinion that the support provided by the primary health care system reduced after a while and identified the viability of digital health interventions to fill this gap. In addition, they felt that the intervention value would increase if it provided some form of opportunity to connect with a health care professional:

Because there is such limited access to midwife and nursing support after having baby now like anything you can access at home...make a difference.

You have lot of contact with the support initially, then you don’t really see anyone.

### Triangulation of Quantitative and Qualitative Data

Triangulating the quantitative and qualitative data provided insights for where key improvements could be made to the app going forward. As shown in Table 8, where these are summarized, these largely focus on improvements that would make the app more valuable to parents.

**Table 8:** Areas to improve and potential improvements.

Areas to improve	Potential improvement
Broadening the scope of the intervention	Intervention could be broadened by including first-aid information and other injury information, including information relevant to a broader age group of children and other early childcare information. Special consideration needs to be given to make the app more valuable for experienced parents.
More autonomy for parents and reframing some advice as “suggestions”	Within intervention content, focus will be given to ensure the advice conveys as suggestions rather than “must follow” advice.
Improving practicality of information	Special consideration will be given to palpability of advice considering a range of individual circumstances of parents.
Improvements to task tracking	The task-tracking feature will be improved by introducing a direct way to access task lists and better ways to identify task ongoing and completion states.
Improvements to if-then plans	If-then tasks will be improved with giving parents a list of options that they can select from to create if-then rules.
Connecting parents to a health care professional	A feature where parents have ≥1 sessions with a health care professional who is experienced in child injury and early childhood could be introduced. This could also be used as a reengaging moment with the intervention for parents.

## Discussion

### Principal Findings

The findings from this study demonstrate promising potential of the intervention in terms of the impact on reducing the risk of infant falls, particularly among new parents. They also indicate promise in terms of an influence on drivers of parental behaviors important for fall reduction among infants. Acceptability of the app was high, and important insights were gained from users about how to further improve the app.

In this study, the potential to reduce the risk of falls was measured by examining the change in responses to a set of questions asked by the participants before and after the 3-month long exposure to the app. As there was no available validated measure, this set of questions was developed on the basis that knowing falls can be prevented, feeling confident that actions can be taken to prevent falls and taking action to reduce fall risk align with what was hoped would be the desired outcomes from exposure to the app. Although this is self-reported and not a validated measure, the relationship observed between this measure and the participants’ responses to questions based on the COM-B self-evaluation questionnaire provides a level of promise that the intervention may work as intended. However, confirmation of the effectiveness of the intervention requires a different methodological approach, such as randomization and use of control, and for this purpose, the use of an objective measure such as reduction in falls would be preferred. Demonstrating the promise of an intervention during the early stages is important, as this reduces the risk of unnecessarily wasting resources in a later, larger, and more resource-intensive randomized controlled trial.

For digital and mobile health interventions, acceptability, usability, and engagement are likely to be as important to effectiveness as the content. The results also demonstrate promise in this regard. Importantly, the users trialing the app appeared to like the app and found it easy to use and useful. More importantly, the feedback from the users identified some areas for further improvement that could be relatively easily actioned, such as improvements to the task-tracking and “if-then” plans, and reframing some of the advice provided to convey more autonomy. However, as identified in user testing during the development phases [13], it was clear from this longitudinal study that in the longer term, the scope of the app needs to be broadened to increase the likelihood of high levels of ongoing engagement.

Concerns raised about the practicality of advice and the relevance of all components of the intervention to all users in this longitudinal study also reflected some of the feedback received during the user testing reported in the study by Cooray et al [13]. As noted in this study [13], issues raised regarding the practicality of advice drawn from best-practice sources indicate a need for further research into practical solutions. However, it may be that it is only certain parents or parents in certain situations who have practical issues, that needs to be explored further. It is possible that the contents of the intervention could be delivered in an individually tailored manner, and this might overcome both the concern of relevance of all information to all users, as well as issues related to the practicality of some advice for certain people or situations. Digital intervention in which injury prevention information is tailored to individuals has been found to be effective in promoting the adoption of safety behaviors relevant to the use of stair gates as well as other childhood injury mechanisms [19]. The potential of this approach should be considered in conjunction with further development of the app.

The potential promise of this behavioral theory–driven app on influencing behavior relevant to falls aligns with the success of other theory-driven digital interventions targeting childhood injury in changing behavior [20-23]. However, this is the first childhood injury intervention developed using the BCW. The significant association between the “behavior score” calculated from responses to the parental COM-B self-evaluation questionnaire and the outcome measure observed in this study also appear to be the first attempt at examining the pathways through which a behavioral theory–driven childhood injury prevention intervention works. Although the approach in this study was rudimentary, consideration should be given to designing future rigorous testing of the app in such a way that the mechanistic pathways can be studied in parallel with the overall effectiveness. The quantitative process evaluation being undertaken by Brown et al [24] in conjunction with the evaluation of their user-driven intervention to reduce the misuse of child restraints is an example of how this might be achieved. This level of evidence for the behavioral underpinnings of the success of digital interventions would further strengthen the case for designing childhood injury interventions using a behavioral theory lens.

The person-based approach to app development is likely to have influenced the high levels of usability and acceptance of the app. However, user feedback indicates that more work is required to increase engagement. The importance of engagement in digital injury prevention interventions has also been identified by other researchers. For example, Ning et al [20] cited poor engagement as a factor that may have reduced the impact of their digital intervention on reducing actual rates of injury. In their study, they measured average hours of engagement and felt that the level of engagement was relatively lower in terms of average hours of engagement than had been reported in other successful digital interventions [20]. However, no attempt has been made to directly study the level of engagement and any outcome. In contrast, Burgess et al [22] examined the direct association between their measure of engagement and an increase in knowledge and found a significant association. Including objective measures of engagement in future attempts to quantitatively evaluate processes underpinning the success of digital interventions would also appear to be useful.

The lack of an objective or quantifiable measure of engagement in this study was a limitation, and tracking engagement is something that should be added to the protocols of any future studies with this app. Other important limitations of this study are as follows: the use of an unvalidated outcome measure for the performance of the app and the pre-post design, which means no causal relationship between exposure to the app and the outcome measure can be confidently claimed. Furthermore, when reviewing the results of this study, it should be noted that recruiting participants via an internet-based panel means that the sample is possibly biased toward inclusion of only those who are already digitally active and computer literate, which may affect the generalizability of the findings. Future studies should aim to assess the intervention across a broader segment of the population. Keeping these limitations in mind, a strength of the study lies in the usefulness of the work as an intermediate step between optimization through user testing and a more resource-intensive controlled trial with a larger population-representative sample.

### Conclusions

The 3-month longitudinal user-testing study has demonstrated the potential promise of the behavioral theory–driven, person-based intervention and has highlighted further scope for refinement. Overall, broadening the scope of the app appears to be the most important issue to be addressed in future work.

## Appendix

Multimedia Appendix 1 App screenshots.

Multimedia Appendix 2 Interview discussion guide.

## Abbreviations

BCW: Behavior Change Wheel
COM-B: Capability, Opportunity, Motivation–Behavior
