# Supplementary figures and images for: Exploring the Potential of a Behavior Theory–Informed Digital Intervention for Infant Fall Prevention: Mixed Methods Longitudinal Study
Source: JMIR Pediatr Parent. 2024 Jan 3;7:e47361. doi: 10.2196/47361 (PMC10794959; doi:10.2196/47361)

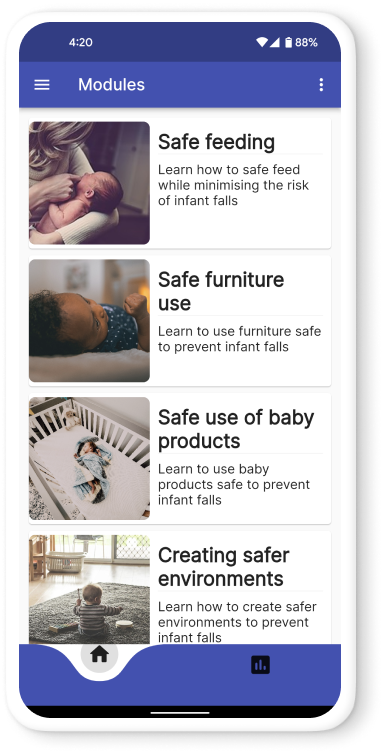

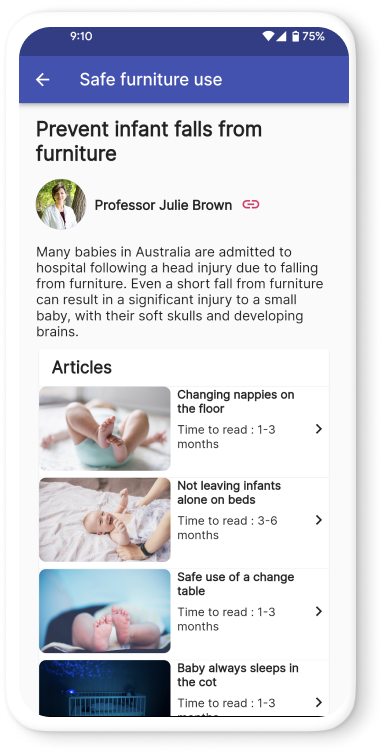


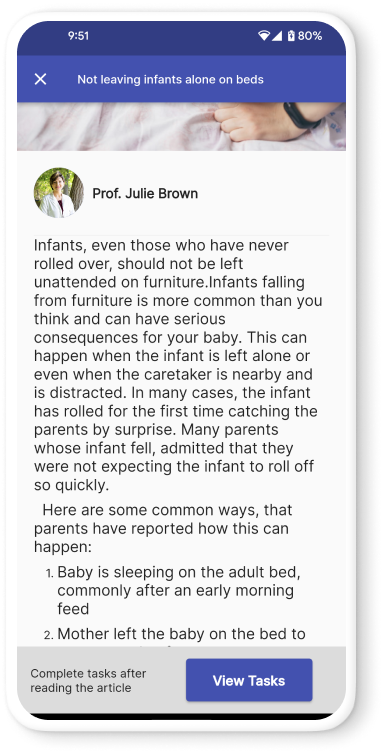

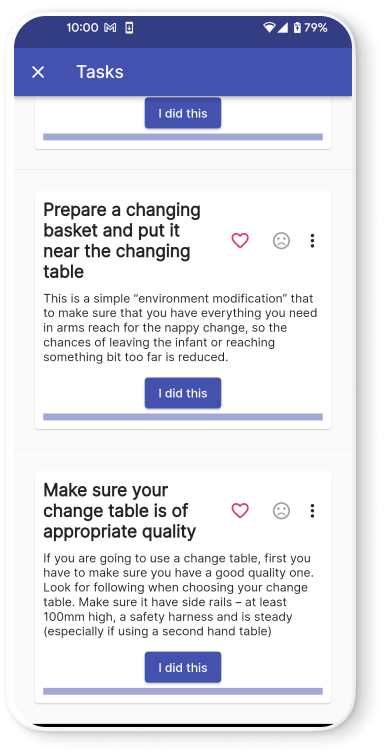


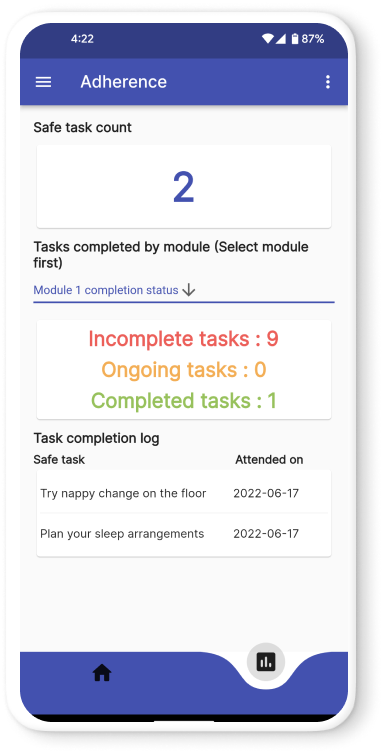

Supplement: Multimedia Appendix 1 [file pediatrics_v7i1e47361_app1.docx]
