# Supplementary material for: Exploring the Potential of a Behavior Theory–Informed Digital Intervention for Infant Fall Prevention: Mixed Methods Longitudinal Study
Source: JMIR Pediatr Parent. 2024 Jan 3;7:e47361. doi: 10.2196/47361 (PMC10794959; doi:10.2196/47361)
Supplement: Multimedia Appendix 2 [file pediatrics_v7i1e47361_app2.docx]

Multimedia Appendix 2

**Interview protocol to explore intervention effects on target behaviours and user experience of the app**

Hi My name is [Name of the researcher].

Thank you for giving us time for this interview.

As you know, the goal of this app is to help parents to prevent fall related injuries, that can happen to babies.

And using these interviews, we are trying to understand how this was received and how to improve the app and getting your honest feedback is a really important part of it.

I’ll ask you set of questions in this interview, there are no right and wrong answers.

[Quick layout of the interview]

**Context questions**

Can I start by asking your thoughts on using apps and websites for early childhood information and baby care… and what are the ones you use/have used? (and that is for…).

Did any of those include information on child injury or before using THIS app, what did you know about child injury in Australia? And where did you get that information?

Now let’s look at our app. Feel free to look at the app and even skim through the app, if that helps when answering the next questions.

**Questions to understand parents’ experience with the app**

1. What do you think about the information provided within the app? (prompt : Did you read all the articles?)
2. What do you think about tasks and tracking them and your thoughts on the concept of dashboard with summary information?
3. How do you find the notifications, that was sent?
4. What do you think about the aesthetics of the app i.e. colours, text sizes, use of graphics?
5. Was there anything you particularly liked (app feature/information)?
6. Was there anything you particularly disliked (app feature/information)?
7. Any suggestions on how we can improve these features? (articles, tasks, notifications)

**Questions about their experience with safe behaviours**

I’ll now ask couple of questions each for 4 “modules” and the information provided.

About 1: Safe feeding

1. Thinking about getting enough rest with your new-born [possible prompts: how did the app impact you, help you received from others?]
2. What was your experience with the suggested feeding practise – Prepare, position, place [possible prompts: How was your experience with this and did you felt this is something you should do?]

About module 2: Safe furniture use

1. How did you find not leaving infant alone on bed [possible prompts: How did you feel about this and is this something you would always do?]
2. (How do you find advice on) Using the cot [possible prompts: what is the baby’s usual sleeping place?]
3. How did you find the information on nappy changing practises [possible prompt: how did you change nappies currently?]

About module 3: Safe use of baby products

1. Do you think you use safety straps regularly? [possible prompts with all products,]
2. Do you have/use a baby walker?

About module 4: Creating safer environments

1. Safety gates and stairs [possible prompts: Do you have stairs in your house? If yes, the practise of safety gates and making stair safer]
2. Overall, do you think this is something you would have used, if come across outside of this study [Yes/No[, [If yes – can you explain why] [If no – why?]. From where this app should be introduced to you? (Prompt: parenting class, kidsafe)
3. Do you have any suggestions for how the app could be improved? [To make it more valuable to parents]
4. Are there any other comments you would like to make?

[Ending remarks]
